# TRAINING RESEARCHERS TO CREATE RESEARCH ADVISORY BOARDS WITH OLDER ADULTS

**DOI:** 10.1093/geroni/igac059.1277

**Published:** 2022-12-20

**Authors:** Rachel Lessem, Rebecca Berman

**Affiliations:** CJE SeniorLife, Chicago, Illinois, United States; Leonard Schanfield Research Institute, Chicago, Illinois, United States

## Abstract

The Sage Resource Project’s goal was to prepare researchers to use the Sage Resources to create Sage Model boards in order to continue to expand and amplify the voice of older adults in aging PCOR/CER research. Training researchers is essential to amplify the voice of older adults in research. This project culminated in the creation of a training manual as well as critical lessons learned for the advancement of engaged research with older adults receiving Long Term Services and Supports (LTSS). After developing a series of webinars for national dissemination, 5 research centers were identified as potential sites for intensive and tailored training on The Sage Model and, ultimately, two research centers completed the training with quite different approaches and results. This session will review the training that was developed as well as the lessons learned from training researchers on creating research advisory boards with older adults.

